# Perspectives in Veterinary Pharmacology and Toxicology

**DOI:** 10.3389/fvets.2016.00082

**Published:** 2016-09-13

**Authors:** Arturo Anadón

**Affiliations:** ^1^Department of Toxicology and Pharmacology, Faculty of Veterinary Medicine, Universidad Complutense de Madrid, Madrid, Spain

**Keywords:** veterinary pharmacology and toxicology, antimicrobial resistance, contaminants and residues, environment, regulatory aspects, new challenges

Veterinary pharmacology and toxicology are being increasingly recognized as important disciplines and were also rapidly changing and evolving in the mid-twentieth century. By the mid-1950s, pharmacology and toxicology were a highly active area of veterinary medicine; veterinary pharmacology and toxicology emerged as new disciplines, closely related, because they were involved in developing new compound, particularly new antimicrobials and antiparasitics to control infection diseases, and analgesic/non-steroidal anti-inflammatory drugs affecting different animal species. The research and development cost of new antimicrobials that must fulfill the new regulatory requirements is considered high; in drug development projects, toxicology closely linked with pharmacology and pharmacokinetics is decisive in the success or failure of a new drug candidate. During the last years, the following new antimicrobial active substances have been authorized for use in veterinary medicine: difloxacin (belonging to the class fluoroquinolones, authorized in 1998 after marketing of enrofloxacin in 1987), valnemulin (a pleuromutilin, 1999), pirlimycin (a lincosamide, 2001), tulathromycin (a macrolide, 2003), tylvalosin (a macrolide, 2004), ceftiofur (a third-generation cephalosporin, 2005), cefovecin (a third-generation cephalosporin, 2006), gamithromycin (a macrolide, 2008), pradofloxacin (a fluoroquinolone, 2011), and tildipirosin (a macrolide, 2011). None of these antimicrobials belongs to a new class of antimicrobials. It is known that most veterinary drugs derive from either the human pharmaceutical industry (antibiotics, analgesic, antiobesity, anticancer, cardiovascular) or the plant protection products (insecticides, parasiticides). Like veterinary medicine, veterinary pharmacology and toxicology is not an easy matter because of multiplicity of animal species as well as breed, age, sex, and (patho)physiological status concerned that may respond differently to certain drugs, food, feed, pesticides, and industrial products as well as contaminants or toxins. The inter- and intraspecies differences as well the variability within and across animal populations are the essence of veterinary pharmacology and toxicology. It is know the species differences in the metabolism and excretory processes of different chemical compounds. In the 60s of the twentieth century, the extrapolation dose regimens from one species to another was a common practice; however, nowadays it is known that pharmacokinetics/toxicokinetics (PK/PD) differences remain for drugs, pesticides, contaminants, or toxins that is the cornerstone of veterinary pharmacology and toxicology. Moreover, the analytical techniques to measure plasma/tissue concentrations of xenobiotics are changed and the sensibility been improved. Analytical methods whether intended for use in pharmacokinetic (PK) and metabolism studies, residue depletion studies, or regulatory control programs for residues of veterinary drugs and pesticides, and contaminants, share a common subset of validation criteria. Performance characteristics to be determined for all method validation include specificity, accuracy, precision, limit of detection, limit of quantitation, susceptibility to interference, and information on method calibration. Practicability, applicability under normal laboratory conditions, and ruggedness are the additional criteria for the evaluation of regulatory methods. Residue programs consist of two principal activities: monitoring and surveillance. For monitoring drugs having maximum residue limits (MRLs), animal tissues, such as liver, kidney, muscle, milk, eggs, honey, or fat, are selected. Another group of samples/matrices frequently used are animal feed and drinking water as well as manure, urine, and hair. They are mostly used to monitor prohibited substances and can be taken prior to slaughtering. Analyzing hair has gained some popularity because it has been demonstrated that anabolic steroids can be detected in hair a long time after application of the drugs – that is, when residues cannot be detected any more in urine or manure. It has been suggested that the hair analysis is a strong means to control the illegal use of clenbuterol in animal production, and samples are available from living as well as slaughtered cattle. The thyroid gland is used to monitor the use of thyreostatics; the eye, vitreous humor, or retina (1, 2) have been suggested as tissues for residue screening, such as clenbuterol, ractopamine hydrochloride, and zilpaterol hydrochloride with β-adrenoceptor activity. Hair/feathers (both pigmented and non-pigmented) also show this accumulation effect; the concentration of clenbuterol in bovine brown or white hair was found to be about one-third that in black hair (3).

By the mid-1950s, veterinary toxicology was a highly active area of veterinary medicine. Toxicology was once the science of poisons and intoxication, but currently toxicology should be regarded as the “science of safety” because it is essential in ensuring safety of food, feed, drugs, pesticides, and industrial products used in daily life (4). Biochemical and molecular interactions were discussed, and the tools of other disciplines were brought to focus upon the problems of domestic animal poisonings. An understanding of mechanisms, potential benefits, and risks of antidotes is essential for clinicians who manage poisoned animals. Of the dozens of antidotes currently available, only a few are regularly used. These include activated charcoal, acetylcysteine, naloxone, sodium bicarbonate, atropine, flumazenil, therapeutic antibodies, and various vitamins. Even then, most are used in a minority of poisonings. There is little randomized trial evidence to support the use of most antidotes. Consequently, decisions about when to use them are often based on a mechanistic understanding of the poisoning and the expected influence of the antidote on the patient’s clinical course. Importantly, most poisoned animal patients who reach veterinary clinic/hospital can recover with supportive care alone. In more serious poisonings, decisions regarding antidote use are generally guided by a risk/benefit assessment based on low quality evidence. With it, a new generation of veterinarian emerged. The developing veterinary toxicologist had to understand physiology, pathology, and chemistry, but with extensive knowledge of qualitative and quantitative analytical techniques. Judgment of clinical episodes and knowledge of metabolic and excretory processes were needed. Toxicology becomes intimately familiar with pharmacology and the molecular action of a wide variety of chemicals. Toxicology and pharmacology can be considered as the same discipline on two different ends of spectrum; pharmacology is the study of drugs used at doses to achieve therapeutic effects on an organism, while toxicology is the study of toxicants that produce harmful effect on an organism. Understanding treatments to be administered for specific intoxications was necessary. In conclusion, there are often confusing and confounding assortment of signs, lesions, and analytical results to reach rational interpretations and conclusions for the numerous problems being solved (5).

The veterinary medicine profession was initially focused on domestic animals, particularly those used for food consumption, and horse for transportation. With the growth of more specialized agriculture and production practices, the veterinary profession with its linkage to domestic livestock, stimulated growth of veterinary pharmacology and toxicology. Later on, the veterinary profession was concerned to a growing population of companion animals, including horses (used in equine activities relating to competitive sports and leisure activities), dogs and cats (as integral family members), and other animal species (i.e., will/exotic animals) (6). The antiparasitic, analgesics, antiobesity, cardiovascular, and anticancer drug market for dogs and cats has grown abundantly, which has been the result of the progress of the veterinary pharmacology and toxicology. In both fields of veterinary medicine (i.e., domestic and companion animals, and wild/exotic animals) was strengthened as observation-based medical practice was complemented and, ultimately, supplemented by science-based medicine. During this period, veterinary pharmacologists and toxicologists began to play an important role in veterinary medical diagnostic laboratories (6). With the strengthening of the science base of veterinary medicine, including the quality of the science in the veterinary medical curriculum, the emergence of the comparative medicine character of veterinary medicine became more apparent and was enhanced (7). Comparative Medicine is a distinct discipline of experimental medicine that uses animal models of human and animal disease in translational and biomedical research. Biomedical research included comparative pharmacology, toxicology, and pathology. These changes in the profession were accompanied by increased involvement of veterinarians in research on the species of traditional concern to the profession, domestic, and companion animals (8), and to participation in a broader range of biomedical research activities, involving use of the traditional laboratory animal species, driven largely by concern for human health (7). More and more veterinarians are now conducting pharmacological and toxicological research as their primary mission.

Pharmacology and toxicology are translational sciences. Pharmacology translates fundamental insights into drug action and fate into clinical therapy. Toxicology translates science, transferring knowledge from fundamental science into practical applications to safeguard animal health, human health, and the environment. Toxicology as translational science uses basic and clinical science to solve problems in toxicologically relevant fields. A high level of basic scientific research in toxicology is the prerequisite to assessing emerging risks in the process of developing drugs, chemicals, and materials (9). Nowadays, veterinary pharmacology and toxicology should be regarded as disciplines contributing to the paradigm “One World, One Health,” reducing risk at the animal–human–ecosystems interface.

The development of veterinary pharmacology and toxicology is the same as that for humans, although between human and veterinary medicine, there are differences in size and economic factors (10). The evolution of veterinary pharmacology and toxicology occurred concurrently with evolution of its two roots; the profession of veterinary medicine and the science of pharmacology and toxicology. The veterinary pharmacologist and toxicologists recognize the necessity of considering the various animal species of concern as a main activity (6). Traditional indicators of target organ toxicity used in drug preclinical safety studies consist of a battery of clinical pathology parameters in blood and urine coupled with histopathologic examination of altered tissues, often measured in multidose toxicity studies. In recent years, the expansion of knowledge at the molecular and cellular level has provided the opportunity for considering the addition of a multitudinous of new endpoints to pharmacological and toxicological evaluations (11). Industry and regulatory agencies expect that new and better efficacy and toxicity biomarkers (the latter also known as safety biomarkers) will play an important role in improving key aspects of the drug development process. Successful translation of many biomarkers into clinical practice is now increasing. It is well known to the veterinary clinician, pharmacologist, and toxicologist and to physicians that biomarkers have been used in both human and veterinary medicine for centuries. In some cases, measurement of the biomarkers present in body fluids, urine, or exhaled breath serve as an indicator of exposure or, even, dose of a drug or toxicant. The application of genomics has already proved useful in fundamental research and for gaining a mechanistic understanding of drug activity (12).

New biomarkers of exposure will continue to be proposed. For each potential biomarker of exposure, it will be necessary to conduct experiments to validate the utility of the biomarker. A special challenge relates to recognizing the dynamics of the pharmacokinetics/toxicokinetics of various drugs and toxicants and establishment of quantitative relationships between exposure and dose at any particular time over the course of the intoxication.

The potential list of biomarkers for efficacy and toxic responses is seemingly endless. In all fields of medicine, from different kinds of cancer to various functional diseases of every organ system, new molecular markers are being identified on a regular basis. The challenge for pharmacologists and toxicologists is to consider, from among this array of opportunities, which biomarkers are sufficiently well validated with regard to their linkage to diseases and sufficiently reasonable in cost to warrant their use in exposure–response studies. This includes consideration of the new and highly sophisticated genomic tools. There is a special challenge in designing validation studies to make certain that the experimental design is directed toward identifying specific disease-related endpoints or toxicant-related effects rather than merely being another, albeit more sophisticated, marker of non-specific toxic effects. A serious issue in many previous validation studies has been the use of a single high exposure level and a few short-term observation times. Such studies are unable to evaluate exposure-related changes in biomarkers and may not be able to identify toxicant-specific changes. Most safety biomarkers have been used in safety assessment for many years without being formally qualified, and many of them show deficiencies with respect to sensitivity, specificity, and predictive value (11, 13). With that in mind, during the development of biomarkers, the following steps should be followed: (1) identification, (2) preclinical qualification, and (3) clinical qualification and diagnostic use (14).

Veterinary pharmacology and toxicology is in some countries a unique discipline, but in others is completely split as veterinary pharmacology and veterinary toxicology because of their development and complexity; the veterinary toxicology studies are not only focused in drugs but also addressed other subjects such as food and feed safety, plant protection products, and industrial chemicals among others.

## Veterinary Pharmacology

Veterinary pharmacology is defined as the study of the properties of drugs and all aspects of their interaction with living organisms. Drugs include any chemical agent (other than food) used in the treatment, cure, prevention or diagnosis of disease, or the control of physiological processes (10). The science of pharmacology draws on the knowledge and methods of many allied clinical and non-clinical disciplines, including chemistry, biochemistry, biology, physiology, pathology, toxicology, and medicine.

Veterinary pharmacology is an experimental science dealing with the properties of drugs and their effects on living systems. It has included study of sources of drugs (pharmacognosy), magnitude, and time course of the observed pharmacological effect on the body pharmacodynamics (PD), relationship between administered doses, the observed biological fluid/tissue concentrations of the drug, and time in the body pharmacokinetics (PK), use in the treatment of diseases (therapeutics), and poisoning effects (toxicology).

The residue definition for veterinary drugs in edible commodities of animal origin is obtained from metabolism studies conducted in target species and livestock animals. The metabolites, degradation products, and other transformation products are typically identified and quantified with methods based on the use of substances labeled with radioactive isotopes. Metabolites obtained in these studies are qualitatively compared with metabolites identified in laboratory animals, usually rats, to ensure that substances occurring in significant amounts in edible commodities have been included in the toxicological testing or to determine whether additional testing of individual metabolites is necessary. Metabolism studies in laboratory animals also serve to identify mammalian metabolism.

Drug–drug and drug–feed additive interactions deserve especial attention: a particular reference should be made to side effects and residue formation, and the likelihood of these interactions has to become part of the evaluation procedure. However, the most effective approach to prevent the occurrence of residues is certainly the rationally based selective use of veterinary drugs for which well-educated veterinarians are needed.

The form and the distribution of the residues that result from each authorized mode of application in each species should be determined, and the depletion of the residues from edible tissues or animal-derived foods should be studied. Total residue and metabolism study provides information to establish the appropriate marker residue (is the parent drug or any of its metabolites or a combination of any of these with a known relationship to the concentration of the total residue in each of the various edible tissues at the expected withdrawal time) and to determine the target tissue. A “marker residue” should be identified, which is usually the form of the drug (parent compound or metabolite) that is found at the highest concentration for the longest period in the target food. The tissue in which the highest residues are found is usually designated as a “target tissue” (represents the edible carcass from which residue depletes most slowly and is the edible tissue selected to monitor for the marker residue in the target animal) (15).

The extent to which such differences can be extrapolated to humans should be evaluated; for example, many sex differences in metabolism observed in rats do not occur in humans. The enzymes involved in the metabolism of foreign compounds represent the most important source of interspecies differences and human variability in the biodisposition of the compound and, for many cases, in the generation of toxic effects.

A further important role of such *in vitro* experiments is to investigate similarities and differences between humans and test species in the metabolism and effects of xenobiotics that may provide information critical to the extrapolations normally used in risk assessment.

Veterinary clinical pharmacology is a subset matter of the broad study of pharmacology and is devoted to the study of the clinical effects of drugs on animal patients with a goal of optimizing therapeutic dosage regimens. Knowledge of the PK and PD properties of drugs and their toxic effects is inherent in this veterinary discipline. Clinical pharmacology in the veterinary setting is the clinical discipline devoted to the optimal use of drugs in veterinary patients, maximizing their prophylactic or therapeutic benefits while ensuring that the adverse consequences of drug use are minimized.

Veterinary clinical pharmacology is a clinical science that integrates disease pathophysiology with fundamental concepts of pharmacology to provide a rational basis for drug therapy in animal patients (10). Brown (16) states that the goal of veterinary clinical pharmacology is to apply the principles of pharmacology to more successfully treat animal patients and to more rationally use medications in veterinary medical practice. A fundamental understanding of veterinary clinical pharmacology is essential for good clinicians. The demonstration of efficacy includes the following test phases: (1) description of the mode of action, (2) determination of dose and dosing interval(s), (3) dose confirmation trials, including persistent efficacy trials, and (4) where applicable, clinical field trials. Similarly, knowledge of the pharmacological action of drugs is meaningless unless one also has a basic understanding of the relevant physiology and pathophysiology of the system or tissue adversely affecting the health or welfare of the patient.

There are methods for conducting clinical efficacy studies for infectious diseases and non-infectious diseases. These approaches may include studies in a model of the disease, or alternate methods for evaluating the response to the therapeutic agent in order to get utility in the new animal drug development process (16). The choice of the clinical endpoint is critical and determines the study design. There are many compounds (e.g., antibacterials, anthelmintics), where efficacy endpoints are reasonably easy to identify (i.e., clinical and bacteriological response as determined by the use of appropriate clinical, postmortem, and bacteriological diagnostic methods, and the determination of mortality rate; decrease in worm count, decreased temperature). However, identification of efficacy endpoints for non-antibacterial or non-growth enhancement compounds is more difficult to determine. Veterinary pharmacology aids in the identification of appropriate endpoints, helping to make them more quantitative, reproducible, and representative of the clinical field situation. Whereas “therapeutics” is a term describing treatment of disease in general and includes use of drugs, surgery, radiation, behavioral modification, and other modalities. Demonstration of efficacy of veterinary drugs is usually dealing with general requirements for the assessment of efficacy of such products. Depending on the aim of the trial, it can be classed in one of the following three categories: (1) confirmatory, (2) exploratory, or (3) composite trial. Confirmatory trials can concern dose-determination trials, dose confirmation trials as well as controlled field trials.

For antimicrobials, the dose, the dosing interval, and the number of administrations of product should always be justified by considering the PK/PD relationship, if established, as well as the severity of the disease. In some cases, where the PK/PD relationship is well established using validated models, it may be possible to omit dose-determination studies and to evaluate in a clinical trial the efficacy of one or a very few regimens. However, to be acceptable, the choice of the PK/PD parameter considered as best predictive of efficacy must be prospectively justified by independent data.

A prerequisite for clinical studies is to perform toxicological testing of new drugs with the objective to know potential undesirable effects of the drug candidates and drugs in the therapeutic range and above (17).

## Pharmacokinetics/Toxicokinetics

Pharmacokinetics (PK) is the study of the characteristics of the time course and extent of drug exposure in individuals and populations and deals with the absorption, distribution, metabolism, and excretion (ADME) of drugs. PK is defined as the mathematical description of temporal changes in concentration of drugs within the body (18); such studies provide the experimental basis for drug dosage regimens in various animal species.

Bioequivalence is a clinical term referring to formulations of a drug with rates and extents of absorption that are sufficiently similar that there are not likely to be any clinically important differences with respect to either efficacy or safety. In order to demonstrate bioequivalence for systemically active drugs, a comparative PK study is generally undertaken and the similarity (defined by statistical and biological criteria) of Cmax (maximum concentration) and AUC (area under curve) of the formulations is assessed. For drugs not acting systemically, comparisons of clinical or other pharmacological endpoints may be necessary.

In the development of dosage forms, there are many factors that must be standardized in order to ensure that the biological availability of the active constituent is both predictable and reproducible. The following physicochemical and formulation-induced factors have been described as sources of potential bioequivalence between products.

Toxicokinetics (TK) relies on the same principles as PK, but there are differences in data availability and aims. The exposure environment is characterized by the concentration of the toxicant in the media, be it water, air, or feed, the quantities taken in, and the time course of the intake. Dose is the concentration, over time, of the toxicant in the various tissues of the subject (cow, human, rat). The characterization of the kinetics linking exposure with dose is referred to as TK (for a toxic agent) or PK (for a pharmaceutical). In actual practice, the term pharmacokinetics is frequently used when it would be more appropriate to use the term toxicokinetics. Toxicokinetics are used to describe the movement and disposition of the toxicant in the organism. This includes consideration of the route of entry: ingestion, inhalation, dermal, or purposeful administration by injection. A complete description of the toxicokinetics of a toxicant will take into account (1) the intensity and duration of the exposure, (2) the rate and amount of absorption of the toxicant from the site of entry, (3) the distribution of the toxicant within the body, (4) potential biotransformation to less, equal, or more toxic form, and (5) the rate of excretion by route (urine, feces, or exhalation). All of these aspects of toxicokinetics may be influenced by species differences in physiological and biochemical characteristics. Modern approaches to modeling toxicokinetics attempt to take account of both species differences and similarities in influencing the fate in the body of toxicants. It is also important to recognize that the exposure or dose level may influence the kinetics of a toxicant and its metabolite(s). This is an especially important consideration in extrapolating from laboratory studies that may be conducted at high doses to lower more environmentally relevant exposures/doses.

## Pharmacokinetic/Pharmacodynamic Predicted Indexes

A PK/PD model is a mathematical description that provides clinically relevant information about the relationship between the pharmacokinetics and the pharmacological effect. There is a mounting body of evidence to support the use of PK/PD relationship for individual antibacterial agents in identifying those doses and dose intervals that are most likely to be efficacious and least likely to result in adverse effects and/or the selection of resistant organisms. This concept has implications for the safety and efficacy of antibacterial drugs (19). PK/PD modeling is a scientific tool to help developers select a rational dosage regimen for confirmatory clinical testing. A PK/PD model integrates the PK model (describing the relationship between dose, systemic drug concentrations, and time) with the PD model (describing the relationship between systemic drug concentration and the effect vs. time profile) and a statistical model (particularly, the intra- and interindividual variability of PK and/or PD origin). PK/PD concepts can be applied to individual dose optimization (20). Aspects of PK/PD relationship through concept, basic theory, and practical data reinforced on data analysis and modeling and simulation, and a chance to play an active role in analysis, interpretation, and discussion will be welcome. Use of the PK/PD relationship can be made to justify the dosages to be used in dose-determination studies. In some cases where the PK/PD relationship is well established using validated approaches, it may be possible to omit dose-determination studies and to confirm the efficacy of one or a very few dose regimens in clinical trials (dose confirmation and clinical field studies). To be acceptable, justification should be provided prospectively for the eligibility of such an approach (19). In conclusion, an effective antimicrobial should possess both PK and PD selectivity; it should distribute in the locus of the targeted pathogen, and it should have no PD impact on comensal microbiota of the gastrointestinal tract of the treated animal or on environmental ecosystems. Presently, we need to revise dosage regimens of the antimicrobial classes that are in the market and use preferentially narrow spectrum antimicrobials.

Veterinary drugs cover a broad range of chemical and class structures and usually undergo metabolism after any route of administration to an animal species. There are species differences in transformation and excretion on several drugs being the half-live values different. Modes of administration include injection, implantation, dermal application by spray or *pour-on*, and inclusion in feed or water, all of which may result in different rates of absorption, with possible differences in the tissue distribution and nature of the residues. Finally, as veterinary drugs are applied to a diverse group of target animals differing in species, breed, age, sex, and (patho)physiological status, the biological variance deserves to be given more attention not only in the developmental stage of new compounds but also at the stage of drug application by the practitioner (20).

## Veterinary Toxicology

Veterinary toxicology is a very complex subject as it deals with a wide variety of poisons. The field of toxicology is very broad, including the identification and characterization of a number of toxicants (pharmaceuticals, food and feed additives, natural toxins, consumer products, and specific chemicals), their physical and chemical properties, their fate in the body, and their biological effects. Currently, synthetic compounds constitute the largest number of chemicals that are frequently encountered in animal poisonings. Acute poisoning is a common presentation in emergency veterinary medicine. Moreover, veterinary toxicology is concerned with the treatment of disease conditions caused by poisons. Currently, there is a need to integrate veterinary medicine and specifically veterinary toxicology into environmental medicine.

The nature of the toxic responses depends not only on the toxicant but also on the route of exposure, the duration, and intensity of the exposure, and the characteristics of the exposed individual (i.e., species, gender, age, preexisting disease states, nutritional status, and prior exposure to the agent or related compounds) (6). The subject of veterinary toxicology is complicated greatly by the wide variations in responses of domestic, aquatic, wild, and zoo species to toxicants. Of course, there are many other factors that can be involved in the overall toxicity of a chemical. Understanding the complete profile (especially mechanisms of toxicity) of each toxicant is the biggest challenge for today’s veterinary toxicologists (21).

Veterinary toxicology is a multifaceted hybrid that draws on and contributes to the veterinary medical profession, the scientific field of toxicology and, broadly, to medical science. Some have characterized toxicology as a distinct scientific discipline. I view toxicology as an applied area of science addressing important societal issues by drawing on multiple scientific disciplines and professions (6). Veterinary toxicology had a much applied origin, involves the evaluation of toxicosis and deficiencies, identification and characterization of toxins and determination of their fate in the body, and the diagnosis and treatment of toxicoses in domestic animals and companion animals. However, the field has broadened to include concern for contaminants in human food products originating from animals and for contributing to the conduct and interpretation of safety/risk evaluations for pharmaceuticals, food additives, consumer products, and specific chemicals.

The recent worldwide melamine contamination in pet and swine feed indicates the relevancy of veterinary toxicology to current animal health and food safety, particularly on its toxicological aspects. Veterinary toxicology can be challenging because of the low frequency of cases observed in a practice setting. When a toxicosis occurs, it often involves a large number of animals and may also involve litigation (22).

Veterinary toxicologists who understand both normal and disease processes extending from the molecular level to the integrated mammalian organism and, indeed, populations, have an array of opportunities for making significant contributions to society (6).

In veterinary medicine, the discipline of clinical toxicology concerns the protection of companion, exotic/wildlife, and food-producing animals. Veterinary clinical toxicology links preventive and experimental toxicology with clinical veterinary medicine. Clinical animals are exposed to natural toxicants in their native environments, as well as to synthetic chemicals and drugs. The practicing veterinarian and the diagnostic or clinical veterinary toxicologist are often the first to respond to these problems, which may appear as a clinical syndrome of unknown cause. In veterinary medicine, understanding of sources of poisons, circumstances of exposure, diagnosis of the type of poisoning, treatment, and application of management or educational strategies to prevent poisoning is of much interest. The clinical diagnosis may be used initially to determine the organ systems affected, and the disturbances that must be controlled to save the animal’s life and etiologic diagnosis is the most important diagnosis because it enables specific therapy and preventive measures to proceed. Clinical toxicology evaluation depends heavily on the determination of exposure and evidence for the contribution of interacting factors than can alter toxicity (23).

## Regulatory Pharmacology and Toxicology

Regulatory pharmacology and toxicology is the study of efficacy and adverse effects of chemicals not just on humans but also on all living organisms, including natural toxins, animals, fungi, and insects. Referring to regulatory toxicology supports the development of standard protocols and new testing methods to continuously improve the scientific basis for decision-making processes. It is the duty of the industrial pharmacologist and toxicologist to aid in the application, interpretation, and circumstantial evaluation of laws, rules, and regulations. Extensive regulatory and monitoring systems have been established to ensure that chemical residues in food do not constitute an unacceptable health risk. Chemicals (i.e., veterinary drugs, medicated feed, or application of pesticides) are everywhere, in the food we eat and in the feed of animals. Regulatory authorities establish MRLs or tolerances and set withdrawal periods that ensure residues of the pharmacologically active substance will not exceed the MRL when the label instructions for the product are followed (15). Some chemicals are used to help people and animals, such as the chemicals in medicines. Surveillance programs by comparison, sample target-tissues from animals suspected of violative residues on the basis of clinical signs or herd history. Animals identified with violative residues of veterinary drugs or pesticides do not enter the food chain. Most people know that high doses of some chemicals used to help them in medicines can damage health. The same can occur in the animals (22).

Veterinary toxicologists try to find out (1) what the toxic effects of chemicals are; (2) where toxic effects occur; (3) how a chemical causes its toxic effects, and (4) what length of exposure causes a toxic effect. Veterinary toxicologists often find out about the effects of chemicals by collecting data from laboratory experiments on animals. They use their skills to predict what is likely to occur if humans come into contact with the same chemical. In laboratory studies, animals are often exposed to much higher levels of a chemical than would normally be experienced by humans from normal use of that chemical. Toxicologists have developed a number of ways of predicting the likely effects at lower exposure levels.

The previous strong emphasis on field observations was first complemented and then supplemented by experimentation. This, in turn, led to the current strong mechanistic orientation of toxicology. With this shift in toxicology came an increased awareness of the utility of a comparative medicine orientation in research directed primarily toward improving human health (7). With this comparative medicine orientation came increased opportunities for individuals educated in veterinary medicine, including veterinary pharmacology and toxicology, to contribute to general pharmacology and toxicology and biomedical science.

## Medicinal Products Intended for Minor Use or Minor Species

There is considerable concern in the animal health sector about the lack of authorized veterinary medicinal products for minor uses and for minor species (MUMS). A “minor use” within EU member states may be a major use in other parts of the world where climate, diseases and disease vectors, environment, and husbandry practices differ from those in the EU. All other animal species that are not considered major [i.e., *major food-producing species*: cattle (dairy and meat animals), sheep (meat animals), pigs, chickens (including laying hens), and salmon; *major companion animal species*: cats, dogs]. All other animal species, which are not considered major, are as a consequence, by default, classed as minor species. Minor use in a major species is generally considered as the use of veterinary medicinal products for the treatment of diseases that occur infrequently or occur in limited geographical areas and thus are indicated for a smaller market sector. The policy is intended to stimulate the development of new veterinary medicines for minor species and for diseases occurring infrequently or in limited geographical areas in major species that would otherwise not be developed in the current market conditions. For these reasons, the published guidelines are intended to reduce data requirements, where possible, for products classified as MUMS/limited market while still providing assurance of appropriate quality, safety, and efficacy and complying with the legislation in place and leading to an overall positive benefit–risk balance for the product (24). Studies on comparative metabolism may be of particular importance when assessing MRL recommendations for multiple species, particularly for the extension or extrapolation of MRLs to minor species. Where identical or very similar MRLs have been set for three major species from different animal classes (ruminants, monogastrics, and poultry), based on specific residue data, confirming a similar exposure situation of the consumer in relation to these species, it can be assumed that the exposure assessment and consequently the risk characterization on the basis of same/similar MRLs for further species beyond the animal classes concerned would be similar. The MRLs for chicken may be extended to turkeys based on similarity between the species (24).

## Toxicity Tests

Toxicity test on laboratory animals are conducted to evaluate chemicals for their potential to cause cancer, birth defects, and other adverse health effects. Information from toxicity testing serves as the basis for regulatory decisions concerning toxic chemicals. Current test methods were developed incrementally over the past 50–60 years and are conducted using laboratory animals, particularly rats and mice. Test animals are often exposed to higher doses requiring assumptions about effects at lower doses or exposures. Often controversial uncertainty factors must be applied to account for differences between test animals and humans (25). The visional strategy for the replacement of animal testing by alternative approaches is based on the “3Rs principles” (Reduction, Replacement, and Refinement), which have been encouraged in particular in Europe. These principles have influenced the modern state and perspectives of the development of non-animal experimental methods. While full replacement has not been achieved yet for those toxicological endpoints, partial replacement strategies to reduce the number of animals used exist. The term “alternative of animal testing” is generally associated with the “3Rs principles,” and was suggested due to animal welfare concerns (26).

*In silico* methods and *in vitro* systems are traditionally much used approaches to complement or partly replace animal tests in pharmacology and toxicology (26). The “integrated test strategies,” which combine *in silico, in vitro*, and *in vivo* methods, allow the best predictions of toxicity.

Predominantly, predictive toxicological testing is derived from the findings to assess risks to animal, humans, and the environment. While traditional toxicology methods have relied heavily on animals, new high-throughput screening approaches to generate toxicological data are becoming increasingly available for the safety assessment of chemicals. Gene expression profiling lends itself to two highly topical areas of drug development: “predictive” toxicology and mechanism-based risk assessment. The last few years have seen a lot of progress being made in linking the profiles of gene expression induced by drugs with their toxicity (27).

Two recent reports prepared by Committees of the National Research Council provide insight into how some individuals envision the future of toxicity testing. The first NRC Report (28) addresses the application of toxicogenomic technologies in predictive toxicology and risk assessment. The second report provides a strategy and vision for toxicity testing in the twenty-first century (25). Both committees would have benefited from participation by veterinarians who understand the complexity of disease processes and toxicoses to compensate for the view of molecular and cell biologists who focus on the microlevel and systems biology and may overstate the role of mathematical models (6). The term systems biology (biological integration) is a realization that organisms do not consist of isolated subsets of genes, proteins, and metabolites. It uses an integrated approach to study and understand the function of biological systems, and how perturbations of such systems (29). The key elements in systems biology are bioinformatics and mathematics that enable data integration and interpretation. Genomics and systems biology had a impact on various aspects of pharmacology, toxicology, and therapeutics; so approaches based on genomics and systems biology are now being used in various stages of drug discovery and development (30).

The concept of systems biology has come to the forefront in recent years. The concept of a systems approach to normal biology and disease processes has been a fundamental concept undergirding both veterinary medicine and human biology.

## Pharmacogenetics/Toxicogenomics

The linkage between dose and adverse health outcome involves multiple mechanisms as various toxicants may potentially impact all the cells and organ systems of the body. Increasingly, scientists have attempted to model these relationships, which, in parallel to the nomenclature for the kinetic phase, are called toxicodynamics (TD) or PD models. It is obvious that multiple pathways may be involved in a toxicant producing disease and that knowledge of the individual steps will increase as knowledge of basic biological mechanisms increases. For example, the explosion of knowledge of basic biology at the level of the genome (genomics), proteins (proteomics), and metabolism (metabolomics) has provided a basis for exploring the mechanistic basis of toxicant-induced disease with a degree of refinement that could not even be envisioned even a short time ago. Genomics will also improve our understanding of comparative pharmacology and toxicology and thereby the rational use of animals and models (30).

The term genomics is used to encompass transcriptomics, proteomics, and metabolomics (or metabonomics) that describes the formation and fate of mRNA, proteins, and metabolites (30). The development of medium- to high-throughput methods in proteomics, genomics, and metabolomics, together with the revolution in bioinformatics, will lead to the accumulation of vast toxicological information, helping to elucidate bioactivity and mechanisms of toxicity in both *in vitro* and *in vivo* models (31). These approaches will also help in the development and establishment of new biomarkers of effect or exposure. To be more efficient, considerable improvement in the use of novel technical tools (omics-based technology) for effective safety assessment is needed. Omics analysis are now being accepted as essential methodological parts of systems or integrative biology, and it is possible to observe global changes in all RNAs (transcriptomics), proteins (proteomics), or metabolites (metabonomics, also called metabolomics, metabolic profiling, etc.) in cellular systems or tissues. This includes an array of new molecular biomarkers, which have received substantial attention. The omics will allow a comprehensive analysis of biological systems of many different animal species. Determination of the expression levels of multiple gene transcripts, proteins, and metabolites is now feasible and has been termed transcriptomics, proteomics, and metabolomics. Genomics can be used to identify new targets, since it is now possible to analyze changes in the transcription of >20,000 genes in various cell types and tissues in multiparameter experiments. There are two key approaches (1) the detection of cDNAs that correspond to the entire protein-encoding gene and (2) the use of RNAi to determine the relevance of mRNA levels in terms of protein expression and gene function. The transcriptomic data, and how they can be interpreted in the context of the pathology, and other biological data from a toxicology study are of much interest (30).

The toxicogenomics data have begun to be used more widely in the human health assessments of chemicals, and will likely be one of the crucial information sources for next-generation of risk assessment decisions. Given the general familiarity of the toxicologists with high-dimensional transcriptional profiling data and major traditional ways in which such data are analyzed, presented, and interpreted, this course is designed to demonstrate how the toxicogenomics data can become a key element in hazard identification, dose-response analysis, and selection of scientifically justifiable uncertainty factors. By superimposing the opportunities that are now afforded by both traditional and high-throughput genomics data onto the human health assessment paradigm, this course will be informative to the risk assessment practitioners and the toxicology research community and increases the scientific impact of the fundamental toxicology studies.

High-throughput screening (HTS) allows identifying desirable PK properties or compounds with the greatest *in vitro* potency and/or efficacy and/or least toxicity. Development of improved hESCH-based HTS assays for cardiotoxicity assessment; cardiac toxicity is a major concern in drug development.

Toxicogenomics is a mature field, which provides invaluable information on the molecular events preceding or accompanying toxicity; however, most traditional use of gene expression and other ‘omic data in toxicology is largely the same as it was 10 years ago: the mode of action analysis, classification/prediction, and biomarker discovery. The toxicogenomics data have begun to be used more widely in the human health assessments of chemicals and will likely be one of the crucial information sources for next-generation of risk assessment decisions. Given the general familiarity of the toxicologists with high-dimensional transcriptional profiling data and major traditional ways in which such data are analyzed, presented, and interpreted, this course is designed to demonstrate how the toxicogenomics data can become a key element in hazard identification, dose-response analysis, and selection of scientifically justifiable uncertainty factors. By superimposing the opportunities that are now afforded by both traditional and high-throughput genomics data onto the human health assessment paradigm, this course will be informative to the risk assessment practitioners and the toxicology research community and increases the scientific impact of the fundamental toxicology studies. Transcriptional and posttranslational signals are known mechanisms, which promote efficient responses to DNA damage (30). Using HTS assays and small molecule analysis approaches, can be identified translational components of the DNA damage response, which operate through the modification of tRNA. Multiple research efforts are looking to use high- and medium-throughput *in vitro* screening data in identifying chemical hazards. For industrial and agricultural chemicals, research efforts in United States and Europe have characterized the *in vitro* biological activity of chemicals using multiple *in vitro* assays and technologies in order to predict *in vivo* toxicity and prioritize compounds for conventional toxicity testing. For pharmaceuticals, high-throughput *in vitro* screening assays have been integrated early into preclinical drug development to identify toxic compounds and guide medicinal chemistry efforts. In both cases, the application of *in vitro* toxicity screening for prioritization and hazard identification relies on its ability to accurately predict the results of *in vivo* laboratory animal studies and humans. More recently, preclinical testing has been tailored to the specific properties and mode of action of the new drug and may be adapted on the outcome of previous studies (32).

The current objectives of toxicoproteomics in diagnostic toxicology is to define molecular mechanisms of toxicity, screen for drug toxicities, and elucidate biomarkers or signature protein profiles in order to more accurately assess, predict, and diagnose toxicities.

The use of proteomics for diagnostic application and the application of microscopic analyses of feeds and animal ingesta for toxic components in the diagnostic process are presented.

## Veterinary Medicines and Environmental Risk

Advanced and developing societies are increasingly concerned with the impact of veterinary medicines on the environment (33). So, actually, an Environmental Risk Assessment (ERA) shall accompany an application for a marketing authorization for a medicinal product for veterinary use. The assessments are performed in two phases. In the first phase (Phase 1), the potential for environmental exposure is assessed according to the intended use of the veterinary medicine. The Phase 2 assessment is performed in two parts, Tier A and Tier B. Assessment will stop at Tier A if the product has been shown to present no risk. In Phase 1, the likelihood of exposure to the environment is assessed, and if the product does not meet certain criteria (e.g., if the soil PEC is greater then 100 μg/kg), then Phase 2 assessment is required. Phase 2 can be performed in two Tiers, in the first Tier, the likely impact of the substance is assessed using a range of standard tests, and the second Tier involves more detailed investigations into the compartment(s) of interest. A number of groups of veterinary medicines (i.e., fish farm medicines and anthelmintics) are known to be of environmental concern as well as the antimicrobials. After systemic administration, the substances can be metabolized and a mixture of the parent compound and metabolites may be excreted in the urine and the feces, developing in the case of antimicrobials, their selective pressure on microbiota harbored by waste, manure/slurry, and beyond in water and soil. Some 70% of the antimicrobials administered to food-producing animals are excreted as active substances into the environment; furthermore, some antimicrobials are stable in the environment for weeks or months (34). Residues from farms may contain antibiotics and antibiotic resistance genes that can contaminate natural environments. The clearest consequence of antibiotic release in natural environments is the selection of resistant bacteria. The same resistance genes found at clinical settings are currently disseminated among pristine ecosystems without any record of antibiotic contamination. For animals on pasture, the excreta will be released directly to soil, whereas for intensively reared animals, the main route of entry will be through slurry and manure spreading. Although there has long been concern over the use of chemical additives in livestock feed (e.g., potential consequences of insect-free manure), the effects on non-target dung insects and dung dispersal primarily become a concern with some drugs (e.g., macrocyclic lactones). For example, the introduction of doramectin into environment will be intermittent through the field application of sheep manure containing excreted drug residues or by direct defecation by sheep of manure containing doramectin residues. In order to assess the risks posed to the environment by veterinary medicines used to treat livestock, a number of models based on assumptions derived from experience with pesticides, industrial chemicals, and guidelines have been developed for regulatory agencies predicting concentrations of veterinary medicines in soil, groundwater, and surface waters. The potential impact of a veterinary medicine on the environment will be determined by a number of factors, including amount used, usage pattern, metabolism, persistence in manure and slurry, sorption and persistence in the environment, and ecotoxicity. Veterinary medicines can persist in soils for days to years, and studies have demonstrated that half-lives are influenced by a range of factors, including temperature, pH, and the presence of manure.

## Antimicrobials and Anthelmintics Resistance

Antimicrobial resistance (AMR) is the resistance of a microorganism to an antimicrobial drug to which it was previously sensitive. Resistant organisms (they include bacteria, viruses, and some parasites) are able to withstand attack by antimicrobial medicines, such as antibiotics, antivirals, and antimalarials, so that standard treatments become ineffective, and infections persist and may spread to others. Antibiotics have revolutionized the treatment of infectious diseases, but there are public health concerns that efficacy of antimicrobials in man is compromised by their use in animals. Antimicrobial resistence is a consequence of the use, particularly the misuse, of antimicrobial medicines and develops when a microorganism mutates or acquires a resistance gene. This issue is now a significant health problem, each year in the European Union (EU) alone, over 25,000 people die from infections caused by antibiotic-resistant bacteria. Infections due to these selected multidrug-resistant bacteria in the EU result in extra health-care costs and productivity losses of at least €1.5 billion each year. If the current trend is not altered, 300 million people worldwide are expected to die prematurely because of drug resistance over the next 35 years (35).

Antimicrobial resistance spreads through global tourism, transfer of patients between health-care facilities within and from outside the EU, and through trade in food and animals. It is an important global economic and a societal challenge that cannot be tackled by countries or public administrations alone. Therefore, the problem needs a comprehensive “One Health” approach to it. That means that a holistic, multi-sectorial approach involving many different sectors (public health, food safety, biosafety, environment, research and innovation, international cooperation, animal health and welfare as well as non-therapeutic use of antimicrobial substances) is needed to take care of this complex problem.

Antibiotic resistance is also a food safety problem: antibiotic use in feed – for treatment, disease prevention, or growth promotion – allows resistant bacteria and resistance genes to spread from food animals to humans through the food chain. The horizontal transfer of resistant bacteria from living animal to humans has been documented for methicillin-resistant *Staphylococcus aureus* (MRSA) investigated in pigs, cattle, horses, and domestic pets. Although most *S. aureus* infections are hospital acquired, transmission of MRSA from pigs to pig farmers and their families has been documented (36), but these transmissions concerning the livestock MRSA (ST398) seem to be low in humans. Livestock-acquired MRSA in contrast to the hospital-acquired MRSA (HA-MRSA) strains has a very low pathogenicity, and it is hardly transmitted horizontally between humans. The same can be indicated for *Escherichia coli* isolates expressing extended spectrum β-lactamases (ESBLs). The issue of MRSA in food animals is an emerging concern.

Recently, it has been discovered that there is a new mechanism of resistance in bacteria to colistin (caused by the *mcr-1* gene). The gene can be transferred between different types of bacteria, potentially causing a rapid development of resistance. The gene was first identified in bacteria (*Enterobacteriaceae*) in South China (37), and since then has also been found in the EU and other regions. In addition, colistin should be reclassified and added to Category 2 of the AMEG (Antimicrobial Advice ad hoc Expert Group of European Medicines Agency) classification system, which includes medicines reserved for treating infections in animals for which no effective alternative treatments exist. This category includes certain classes of antimicrobials listed by the World Health Organization as critically important in human health, especially fluoroquinolones and third- and fourth-generation cephalosporins, only be used in food animals when their use is justified and under specific restrictions. Responsible (prudent) use of antibiotics is an essential element in the fight against antibiotic resistance.

It is probable that one of the most effective measures to limit expansion of AMR is reducing the need for antibiotics in animal husbandry by improving animal health through biosecurity measures, disease prevention (including the introduction of effective vaccines, modulation of the innate immunocompetence, bacteriophage therapy), and good hygienic and management practices; and eliminating economic incentives that facilitate the inappropriate prescription of antibiotics. More targeted use of antimicrobials were still necessary to guard animal health (e.g., by the use of accurate diagnosis, evidence-based regional treatment guidelines, and correct dosing regimens). It is noticeable that it is necessary to provide improved summary product characteristics (SPC) guidance about the epidemiological circumstances under which this has shown to be effective and the extent of benefit demonstrated. Veterinary pharmacology contributes to make rational dosage schedule design and to minimize its emergence and spread.

There is now a need to develop veterinary medicines, which reduce the need for use of antimicrobials (“alternatives”), such as vaccines, and will facilitate the regulatory pathway for innovative products and novel therapies. Furthermore, there is now a need to develop new compounds having minimal impact on gut flora and on environmental bacterial ecosystems (34).

The development of nematode and trematode resistance to various groups of anthelmintics is a major problem. Compared with the development of AMR, resistance to anthelmintics in nematodes has been slow to develop under field conditions.

## Animal Feed Contamination

In the last years, the animal feed productions have had several contamination crises, such as transmissible bovine encephalitis caused by prions, unexpected sources of dioxin contamination of milk, meat, and eggs (38), and the presence of residues or unauthorized substances, such as melamine in milk, milk products, and animal feeds (39). Feed hygiene is another important subject to be highlighted because of contamination with *enterobacteriaceae* and anaerobes and the presence on environmental pollutants and contaminants, such as persistent organic pollutants (POPs), present in a broad variety of feed material and able to accumulate in animal tissues (i.e., dioxins and dioxin-like polychlorinated biphenyls and brominated flame retardants). Toxic heavy metals and other chemical elements and natural toxins, such as toxic plant metabolites and bacterial and fungal toxins, can be present in feed for animal consumption.

In recent years, increasing attention has been paid to the risk to consumers posed by chemical contaminants or residues in animal feed. This was caused by various cases of milk, eggs, or other animal products contaminated with environmental chemicals. For that, large number of emerging chemical contaminants in feed require the establishment of maximum limits (MLs).

The carry-over or cross-contamination from feed to food must be determined from animal experiments throughout the so-called transfer factors of chemicals from animal feed to animal products. The transfer factor is expressed as the concentration of the chemical in the animal food products (milligram/kilogram wet weight) divided by the concentration of the chemical in the animal feed (milligram/kilogram dry weight). Leeman et al. (40) have compiled data on transfer rates from a number of chemical contaminants in food products [i.e., pesticides, dioxins and furans, polychlorobiphenyls (PCBs), and polybrominated biphenyls (PBBs), heavy metals, mycotoxins, hormones, veterinary medicines, nitrosamines, and other compounds not belonging to one of the previous classes]. If a study on the carry-over is not available, a conservative worst-case estimate considers that all of the contaminant consumed is transferred to the animal product, e.g., the meat.

Carry-over or cross-contamination has been evaluated with some coccidiostats, particularly polyether ionophore. The health risk to non-target species resulting from the consumption of cross-contaminated feed with coccidiostats at levels of 2, 5, or 10% was evaluated recently by the European Food Safety Authority (EFSA) and a revision of the risk assessments is found in the paper of Dorne et al. (41).

To tackle these current and future challenges, we need to advance scientific knowledge through the application of novel approaches. Likewise, for an effective translation of scientific discoveries and applications in the field of veterinary pharmacology and toxicology, we need an outlet to enhance effective communication among scientists with different backgrounds and who share a common interest in veterinary pharmacology and toxicology. This was the drive for launching a veterinary pharmacology and toxicology specialty section in the journal *Frontiers in Veterinary Science*, an international, open-access, peer-reviewed, online journal. It gives me great pleasure to announce the inauguration of this new veterinary pharmacology and toxicology specialty section with the ethos of publishing the most impactful and interesting research discoveries in basic and fundamental areas of pharmacology and toxicology. The veterinary pharmacology and toxicology specialty section will publish original research articles and reviews of latest developments in specific subject areas and forward-looking perspectives.

As the founding Specialty Chief Editor for veterinary pharmacology and toxicology *Frontiers in Veterinary Science*, I have accepted the challenges and responsibilities of developing a premier professional journal in veterinary pharmacology and toxicology. The editorial team will strive to maintain excellence while improving the efficiency of the review process and taking advantage of the latest online technology. All editorial board members are leaders in their respective scientific areas and are willing to devote their time and expertise to build a journal of excellence to serve the veterinary pharmacology and toxicology research community, and life sciences in general, with the ultimate goal of improving animal and human lives. I do hope that the veterinary pharmacology and toxicology section will appeal to readers with veterinary, medical, and life science interests.

## Veterinary Pharmacology and Toxicology is a Specialty Section of Frontiers in Veterinary Science

Veterinary Pharmacology and Toxicology provides an international publication forum for scientists and clinicians investigating drugs and toxins in all animal species (livestock, poultry, game birds, avian, rabbits, companion animals, horses, wildlife, zoo animals, farmed fish and shellfish, bees, and laboratory animals). The section welcomes high quality original manuscripts in all topics of pharmacology and toxicology, including pharmacodynamics/toxicodynamics; pharmacogenetics/pharmacogenomics; toxicogenetics/toxicogenomics; pharmacokinetics/toxicokinetics; adverse effects of drugs, xenobiotics, plants and toxins; contaminants, pesticides, and drug residues in food animals and food products; clinical pharmacology and therapeutics; clinical toxicology; environmental toxicology; AMR; and regulatory pharmacology and toxicology. Veterinary pharmacology and toxicology welcomes submissions in traditional as well as new and emerging areas of research, encourages interdisciplinary studies, and seeks to advance global progress in these important disciplines.

I look forward to receiving your exciting research contributions in these areas. Your ideas and suggestions for making the journal highly impactful are welcomed and will be always valued.

## Author Contributions

The author confirms being the sole contributor of this work and approved it for publication.

## Conflict of Interest Statement

The author declares that the research was conducted in the absence of any commercial or financial relationships that could be construed as a potential conflict of interest.
